# Characterisation of Temporal Patterns in Step Count Behaviour from Smartphone App Data: An Unsupervised Machine Learning Approach

**DOI:** 10.3390/ijerph182111476

**Published:** 2021-10-31

**Authors:** Francesca Pontin, Nik Lomax, Graham Clarke, Michelle A. Morris

**Affiliations:** 1 Leeds Institute for Data Analytics, University of Leeds, Leeds LS2 9ET, UK; N.M.Lomax@leeds.ac.uk (N.L.); M.Morris@leeds.ac.uk (M.A.M.); 2 School of Geography, University of Leeds, Leeds LS2 9ET, UK; G.P.Clarke@leeds.ac.uk; 3 School of Medicine, University of Leeds, Leeds LS2 9ET, UK

**Keywords:** physical activity, unsupervised machine learning, smartphone, secondary data, cluster analysis, data science, big data, self-recorded health data

## Abstract

The increasing ubiquity of smartphone data, with greater spatial and temporal coverage than achieved by traditional study designs, have the potential to provide insight into habitual physical activity patterns. This study implements and evaluates the utility of both K-means clustering and agglomerative hierarchical clustering methods in identifying weekly and yearlong physical activity behaviour trends. Characterising the demographics and choice of activity type within the identified clusters of behaviour. Across all seven clusters of seasonal activity behaviour identified, daylight saving was shown to play a key role in influencing behaviour, with increased activity in summer months. Investigation into weekly behaviours identified six clusters with varied roles, of weekday versus weekend, on the likelihood of meeting physical activity guidelines. Preferred type of physical activity likewise varied between clusters, with gender and age strongly associated with cluster membership. Key relationships are identified between weekly clusters and seasonal activity behaviour clusters, demonstrating how short-term behaviours contribute to longer-term activity patterns. Utilising unsupervised machine learning, this study demonstrates how the volume and richness of secondary app data can allow us to move away from aggregate measures of physical activity to better understand temporal variations in habitual physical activity behaviour.

## 1. Introduction

Physical inactivity is a global health problem, with 1 in 4 adults not meeting World Health Organisation (WHO) physical activity guidance [1]. In their global action plan on physical activity, the WHO has set a goal of a 15% relative reduction in physical inactivity by 2030, recommending a whole systems approach [1]. A key step in tackling physical inactivity is understanding how different patterns in activity behaviour have different impacts on health [2,3]. In the age of smartphones and wearables, the volume and ubiquity of rich individual level physical activity data has never been greater. These data have the potential to provide new insight into physical activity behaviours at both the individual and population level [4].

To date, most of the evidence base regarding physical activity behaviours has come from primary studies; studies which, following a method determined by the investigators, recruit participants and collect specific predetermined self-report or objectively recorded data over a set time period. Primary data collection is often limited by funding and participant burden to small sample sizes and short data collection periods [5,6]. Moreover, this participant recruitment and short-term monitoring has the potential to result in self-selection biases or behaviour modification by the participants, known as the Hawthorne effect or reactivity [7,8]. For instance participants tend to be more active during the initial data collection periods than later in the study [9]. These shortcomings potentially limit insights into habitual and longer-term physical activity behaviours [5]. Participant recruitment also frequently necessitates participants are from the same locality, restricting generalisability of identified behaviours to the wider population [10].

These limitations may play a role in the mixed evidence of seasonal (yearlong) and weekly variations in physical activity behaviour [11,12,13,14,15,16,17]. Few studies to date have looked at physical activity behaviours using daily measures over an extended time period or extensive spatial area [5]. However, smartphone and wearable device data provides the opportunity to unobtrusively achieve this repeated monitoring [18] and reduce participant burden [14,19]. Whilst simultaneously offering the insights that researchers are trying to gain from primary studies. For example, utilising the smartphone data, of over 700,000 users across 111 countries, Althoff et al. observed higher activity, as measured by step count, on weekdays during commuting times and higher levels of activity on weekend afternoons in walkable cities [20]. Furthermore, analysis of data from Strava, a popular app where users record cycling and running activity, has demonstrated seasonal and hourly variations in commuting and recreational physical activity across several countries [21,22,23,24]. Many of the studies utilising smartphone data use secondary data [25], data originally collected for another purpose. These secondary data studies may therefore capture those who do not usually participate in recruited studies and remove the risk of the Hawthorne effect altering participant behaviour [26]. Use of secondary smartphone app data requires careful ethical and data security consideration [27,28]. Additionally the representativeness of any smartphone app user sample must be ascertained from user provided demographic data [26,29]. Still, if used appropriately, the growing market of fitness and health monitoring apps has the potential to revolutionise how we study physical activity behaviour [26].

The volume and dimensionality of individual activity data from smartphones provides methodological opportunities commonly used by data scientists exploring ’big data’ but not yet readily utilised in physical activity pattern analysis [26,28]. Machine learning classification methods are increasingly used to identify activity behaviours from raw accelerometery data, for example identifying periods of walking, running or sedentary behaviour [30,31,32]. Yet these methods can also be applied to detect patterns in activity behaviour themselves. Utilising k-means clustering for example, McConnell et al. identify ten physical activity pattern clusters in data collected from more than 48,000 participants [9]. This unsupervised machine learning approach is advantageous as it allows patterns of activity behaviour to be characterised without relying on prior assumptions [9,33,34]. Moreover, the volume of data these methods afford means it is possible to quantify day-to-day trends in behaviour and longer-term patterns at the individual level, rather than relying on aggregate activity measures. Though these smartphone data may lend themselves to machine learning approaches, care needs to be taken to identify the most suitable clustering approach to identify the physical activity patterns of interest [35].

The aims of this research are two-fold, first to implement two unsupervised methods to provide a robust classification for both annual and weekly physical activity behaviour patterns, as measured by step count. Second, to characterise the demographic and other behaviour characteristics of the identified clusters of physical activity behaviour.

## 2. Materials and Methods

### 2.1. Data

This study utilises smartphone app data provided by Active Inspiration Ltd from their commercial app “Bounts”, which can be accessed by application to the UK Consumer Data Research Centre [36]. The app incentivised physical activity by rewarding users with points for higher activity levels. Accumulated points could be exchanged for prizes, such as gift vouchers, prize draw entries, or merchandise [29]. Data are provided as a daily breakdown of different activity types, with corresponding metrics including activity duration, steps taken and speed. Data are linked with a unique pseudonymised ID to user information including year of birth, gender, and postcode district, where the user chose to enter this information into the app. Cleaned activity data for 30,804 participants who used the Bounts app in 2016 were utilised. The full data cleaning process has been outlined in Pontin et al. [29]. In brief, only users with seven or more days of recorded activity and who entered their gender and age into the app were included in the final 30,804 users [29]. Improbably fast or long activities were also removed. Previous analysis found that although app users were more likely to be female, the Bounts population has a similar socioeconomic profile to that reported for the whole UK population in the 2011 UK Census [29].

#### Data Preparation

Analysis focused on step count as this was the most widely collected metric across activities and captures both app usage and physical activity volume. Moreover, daily step counts have been established as an accessible metric to monitor and set physical activity goals [37]. For each user the number of steps they recorded on each day of 2016 was calculated, with 0 indicating a day where the participant did not record any activity. Step count was capped at 30,000, with daily step counts of 30,000 and above treated equally for the purposes of clustering. This was to ensure variations in the step counts of those who were less active were identified and to ensure that clustering did not only distinguish between the highly active user minority and other users. We investigated patterns in physical activity behaviour within the 30,804 participants both across the year of recorded activity (2016), to detect seasonal changes, and across the weeks of activity to identify commuting and leisure related activity patterns. Users on average recorded 34 weeks of activity across 2016 [29]. To categorise weekly activity behaviour patterns, each week of activity recorded by each user was treated as a separate entity, resulting in 1,059,201 weeks of activity recorded by the 30,804 users (23,933 female, 6871 male) being used in the clustering analysis. Then, 18.6% of activities did not have an associated step count, for instance some gym activities, swimming, and cycling, and activities recorded on some apps, such as Strava, that record distance but not step count if using a phone tracker. These activities without step count were explored further in the cluster interpretation.

All statistical analysis was conducted in Python 3.8. The unsupervised clustering algorithms were implemented using the scikit-learn open-source machine learning library [38].

### 2.2. Clustering Algorithm Choice

The first aim was to investigate the use of a range of unsupervised clustering methods, compare model classifications and assess real-world interpretability. Commonly when evaluating clustering method suitability, algorithms across the different method types are compared, i.e., density, partitioning, and hierarchical methods [30,35]. Algorithm choice was based on those techniques most frequently found in the literature [35]. The agglomerative (hierarchical) clustering and k-means (partitioning) clustering methods, outlined in Table 1, were applied to both the annual and weekly data.

### 2.3. Model Specification

K-means clustering was applied to the yearly and weekly steps data, respectively. The number of clusters was determined using the elbow method, silhouette score, and sensitivity analysis. The elbow method determines the optimal trade-off between number of clusters and minimisation of the value of inertia (within-cluster sum-of-squares), a measure of internal cluster coherence [39]. Alternately silhouette analysis looks to find the trade-off between number of clusters and the maximum average silhouette score, a measure of how similar an object is to its own cluster compared to other clusters [39]. Sensitivity analysis around the optimal number of clusters was used to ensure behaviour patterns were not missed or duplicated between clusters. Agglomerative clustering (hierarchical) was also applied to the yearly steps data and a dendrogram produced. Due to the size of the weekly step data, the data were split into 20 random samples (>50,000 weeks of activity) and the clustering repeated on 5 of the samples the results of the clustering were then compared across samples. In each instance, the number of clusters was determined from observation of the dendrogram, again to minimise cluster number but maximise inter cluster variance [39]. Following interpretation of the clusters, cluster names were assigned, designed to best capture level and seasonal or weekly patterns of physical activity behaviour.

### 2.4. Model Evaluation

Model clustering was compared using the adjusted rand index (ARI) and mutual information score (MI). ARI measures the similarity of cluster assignments ignoring the permutations of cluster labelling and correcting for chance, defined as:

X∖YY1Y2⋯YssumsineX1n11n12⋯n1sa1X2n21n22⋯n2sa2⋮⋮⋮⋱⋮⋮Xrnr1nr2⋯nrsarinesumsb1b2⋯bs

(1) ARI=∑ijnij2−[∑iai2∑jbj2]/n212[∑iai2+∑jbj2]−[∑iai2∑jbj2]/n2

where $n_{ij}$, $a_{i}$ and $b_{j}$ are values from the contingency table above.

MI measures the agreement between clustering assignments, whilst also disregarding labelling permutations with adjusted mutual information (AMI) correcting for agreement due to chance [35,40], defined by the following equations:

(2) $$MI\left(U,V\right)=\sum_{i=1}^{\left|U\right|}\sum_{j=1}^{\left|V\right|}\frac{|U_{i}\cap V_{j}|}{N}\log\frac{N|U_{i}\cap V_{j}|}{|U_{i}\left|\right|V_{j}|}$$

(3) $$AMI\left(U,V\right)=\frac{MI(U,V)-E(MI(U,V))}{avg(H(U),H(V))-E(MI(U,V))}$$

For the weekly activity behaviour MI, AMI, and ARI were calculated between each sample and the k-means clustering classifications, average MI, AMI, and ARI scores were then calculated.

### 2.5. Cluster Characterisation

Visual interpretation of the temporal activity patterns in the defined clusters was used to characterise the patterns observed. For weekly activity behaviours a single user may have recorded activity that falls into several of the different classified weekly patterns. Thus, for each user the proportion of weeks that fell into each cluster was calculated from the total number of weeks that user recorded activity. Demographic characteristics of the clusters were calculated and compared between and across clustering methods. A chi-squared test was used to determine if the gender distribution of each cluster was equal or whether specific clusters had a higher number of male or female users. Additional characteristics of activity behaviour including preferred activity type and meeting of WHO physical activity guidelines, of 150 min of moderate to vigorous physical activity (MVPA), were also compared across weekly behaviour clusters. Some devices use different names for the same activity type, therefore such activities were grouped as outlined by Pontin et al. [29]. For example, checking into a gym, activities recorded using gym equipment and ‘cardio’ workout were all grouped under ‘cardio & workout’ and ‘biking’ was categorised under ‘cycling’. ‘Meps’ were MYZONE Effort Points calculated by Myzone fitness trackers based on activity intensity rather than defining the type of activity.

### 2.6. Cluster Interrelation

For each app user the proportion of weeks recorded belonging to each cluster of week behaviour was calculated and aligned with the individuals’ yearly cluster behaviour. Relationships between cluster membership were visualised to relate weekly behaviour patterns to overall seasonal physical activity behaviours.

## 3. Results

### 3.1. K-Means Clustering Model Specification

**Figure 1:**
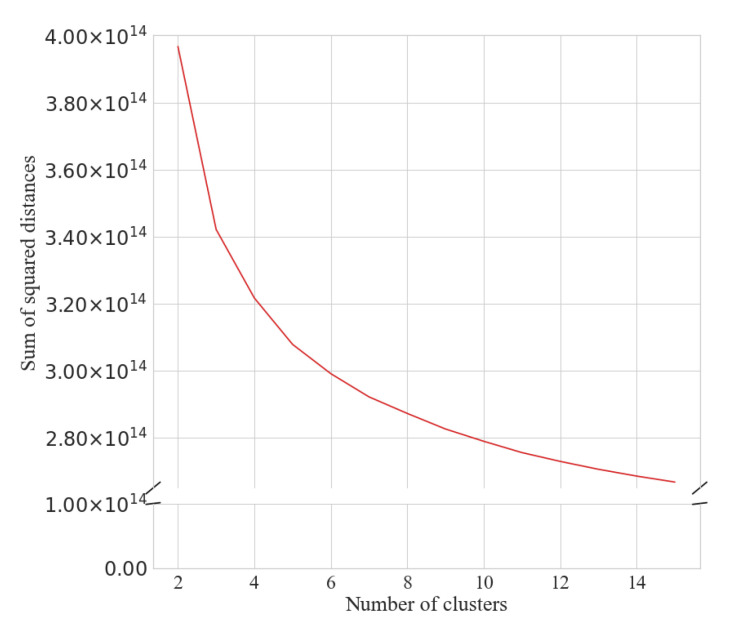
Elbow plot for determining the number of yearlong step behaviour clusters.

**Figure 2:**
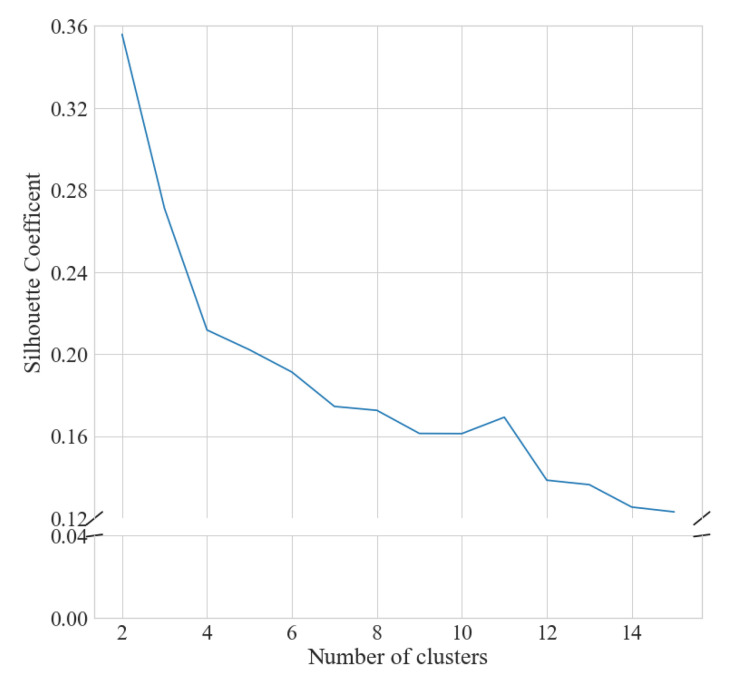
Silhouette analysis for k-means clustering of yearlong step behaviour.

**Figure 3:**
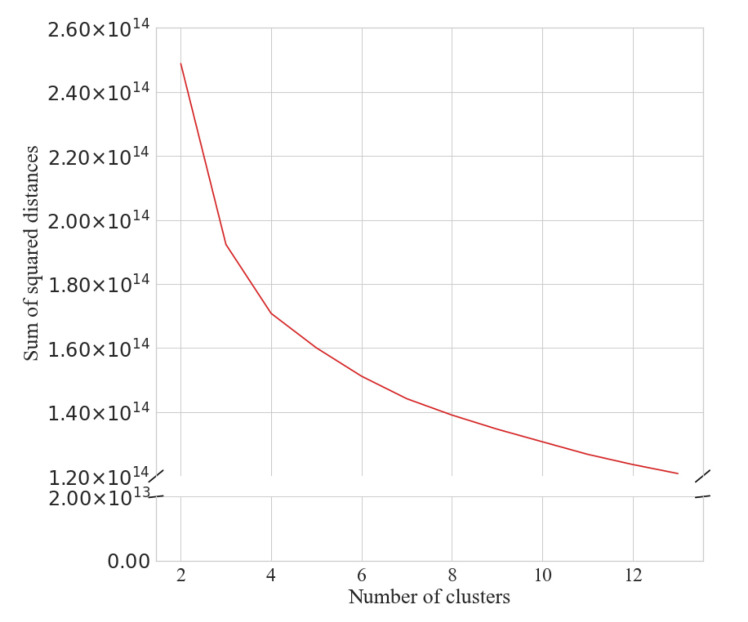
Elbow plot for determining the number of weekly step behaviour clusters.

**Figure 4:**
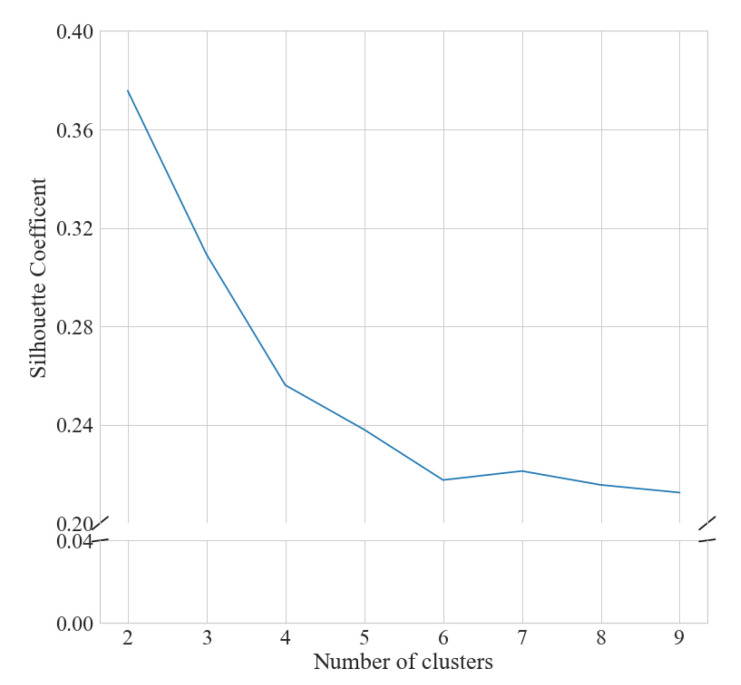
Silhouette analysis for k-means clustering of weekly step behaviour.

**Figure A1:**
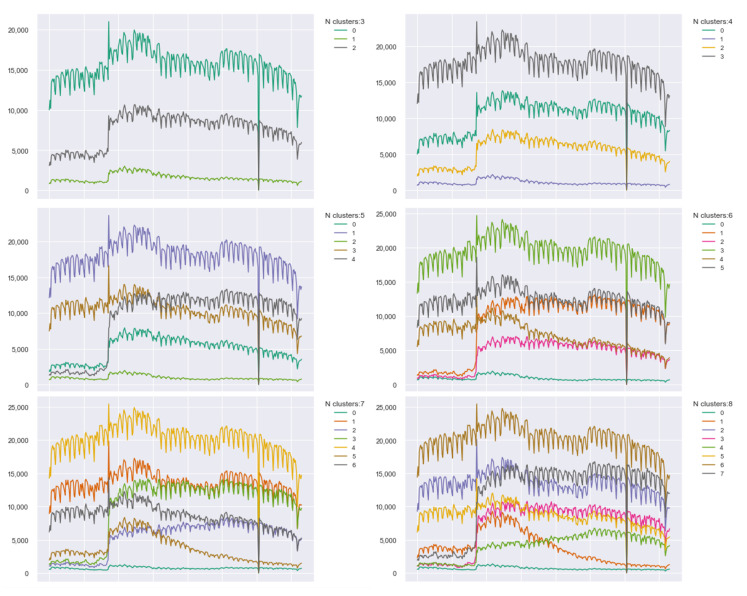
Seasonal k-means clustering sensitivity analysis.

**Figure A2:**
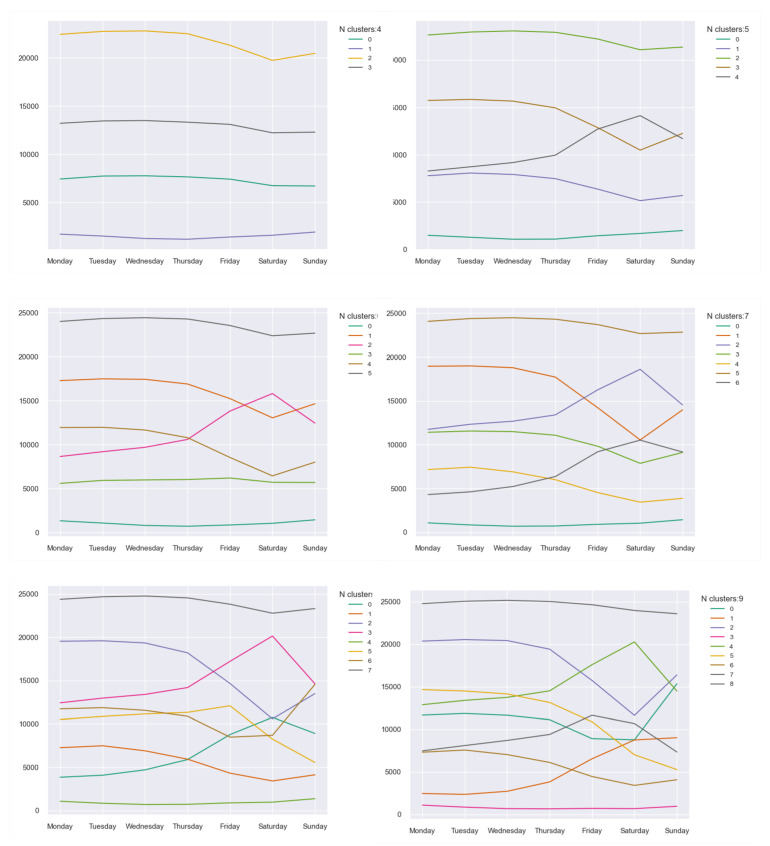
Weekly k-means clustering sensitivity analysis.

The number of clusters was determined by visual inspection of the elbow and silhouette plots for the seasonal (Figure 1 and Figure 2) and weekly (Figure 3 and Figure 4) clustering, respectively. Results of the sensitivity analysis can be found in Figure A1 and Figure A2 and were used to confirm number of clusters. For physical activity behaviour across the year seven clusters were determined as the optimal number that most parsimoniously captured variation in physical activity behaviours. Similarly, for weekly physical activity behaviour six clusters were determined to be optimal.

### 3.2. Agglomerative Hierarchical Clustering Model Specification

**Figure 5:**
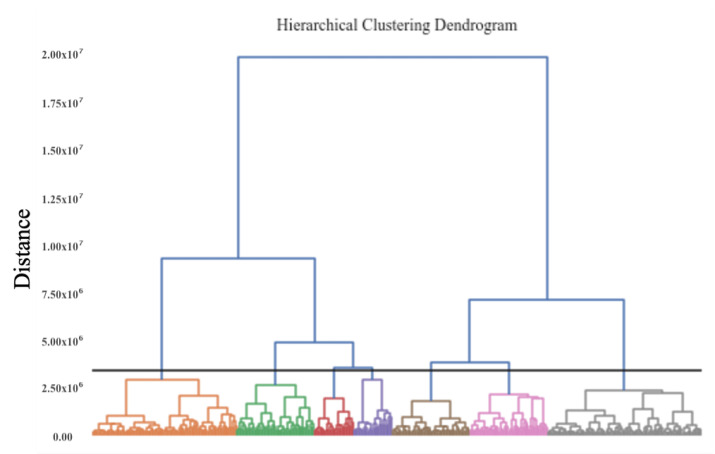
Dendrogram of agglomerative clustering of yearlong activity behaviours, colour indicates final cluster membership.

**Figure A3:**
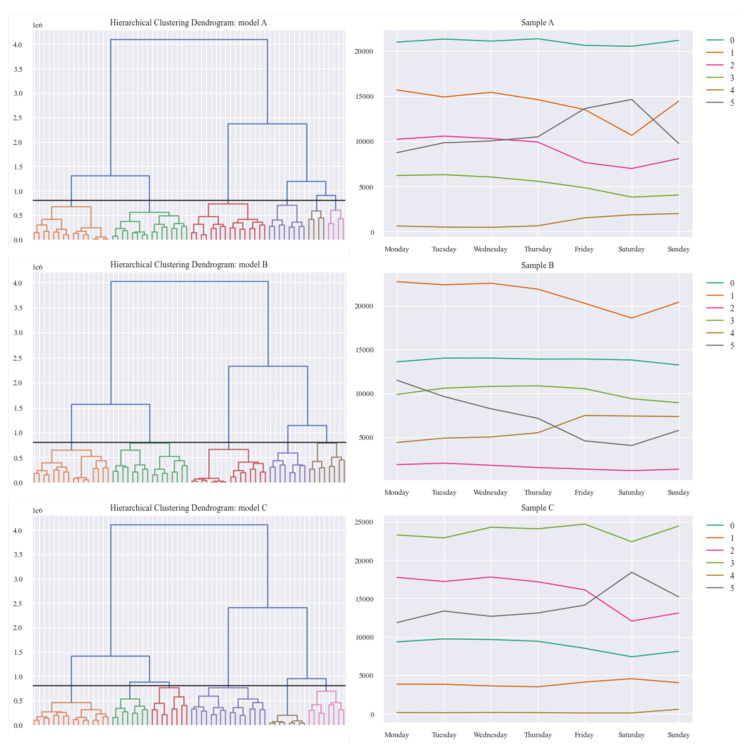

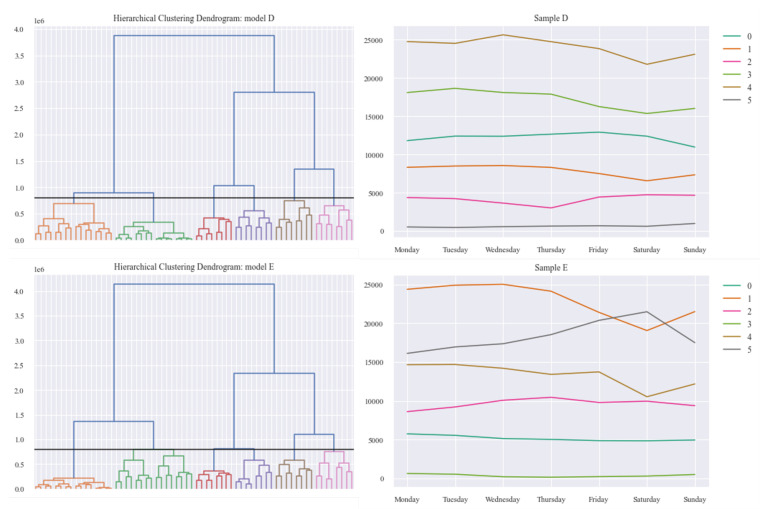
Dendrograms and corresponding activity patterns (N = 6) for agglomerative clustering of the five samples of weekly physical activity behaviour.

For the agglomerative clustering models, the number of clusters was determined by inspection of the resulting dendrogram, to identify the maximum distance between clusters whilst also capturing significant differences in physical activity behaviour. The resulting dendrogram for seasonal behaviour clusters is depicted in Figure 5, indicating seven clusters (coloured) perpendicular to the intersecting black line. Dendrograms for the agglomerative clustering on the five samples of weekly activity behaviour are displayed in Figure A3.

### 3.3. Modelled Behaviours

Due to good agreement between the two clustering methods, we focus on the k-means clusters for brevity in Section 3.3.1 and Section 3.3.2 rather than presenting both. Agreement between the two methodologies is further explored in the model evaluation Section 3.4.

#### 3.3.1. Seasonal Physical Activity Behaviour

**Figure 6:**
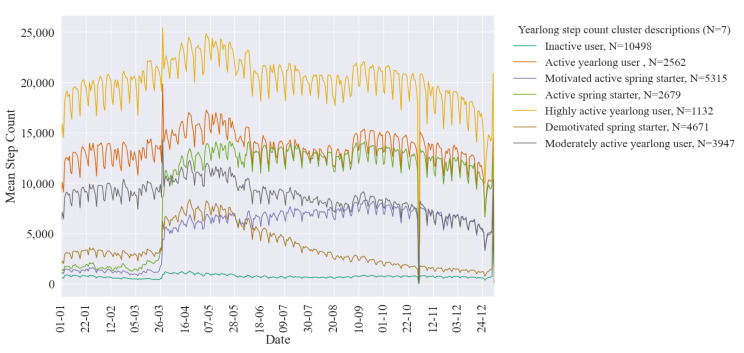
Average daily step counts of yearlong physical activity behaviour clusters (N = number of users assigned to each cluster).

Average daily step count for the assigned k-means clusters is illustrated in Figure 6. As previously reported, UK daylight saving beginning and ending (weeks 12 and 43) have resulted in a misassignment of step count, resulting in an inaccurate peak in March and dip in October [29]. Seasonal patterns in all clusters of activity behaviours are seen, with an increase in step activity following daylight saving beginning, corresponding to lighter evenings and a drop in activity when daylight saving ends, corresponding to less daylight hours for exercise. Cyclical weekly variation is also seen within the clusters across the year. Equally all step activity sees a slight drop off during summer months. Within both agglomerative clustering and k-means defined clusters similar variations in step intensity and behaviour are seen. There is an apparent *highly active cluster* with consistently high step counts throughout the year. Several clusters follow this same yearlong trend in activity as the *highly active cluster* but at varying levels of step count intensity. Around daylight saving beginning we see the emergence of three clusters of users who start using the app at this time. Within these three clusters there are three distinct behaviours; those that start using the app and follow similar seasonal patterns to those who started using the app on or before the start of the year, those who are increasingly motivated and those who start using the app, initially are motivated however seemingly use motivation with falling step count as the year progresses. Finally, an *inactive user* cluster is observed whose step count is consistent low throughout the year. For both clustering methods this low step count cluster, with the highest number of users, was further investigated to look at non-step activities.

#### 3.3.2. Weekly Physical Activity Behaviour

**Figure 7:**
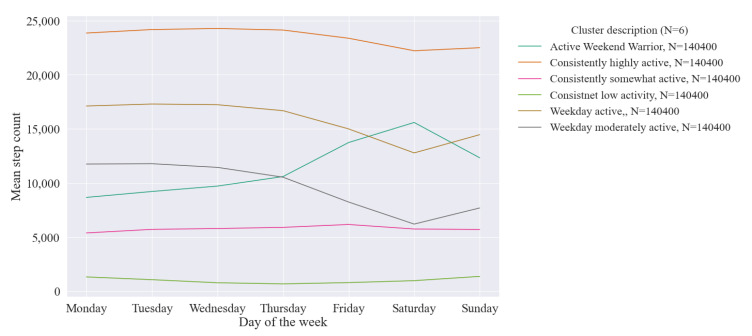
Average daily step counts for k-means weekly physical activity behaviour clusters (N = number of weeks of behaviour assigned to each cluster).

The number of different weekly cluster patterns recorded by users across the year was 3.8, i.e., users on average recorded almost four different patterns of weekly activity behaviour across the year. Figure 7 shows these weekly physical activity behaviour clusters which follow three patterns. First are those whose step count is consistent throughout the week at either a high, moderate, or low step count threshold. Second are the *weekday active*; either averaging below (moderately active) or above (active) 10,000 steps a day. Finally, we see the *‘weekend warriors’* who have a much higher step count on the weekend than weekdays. The less active clusters (with a lower step count) are the larger clusters, i.e., the more common activity behaviours, with the *consistently highly active* cluster having the lowest membership.

### 3.4. Model Evaluation

**Figure 8:**
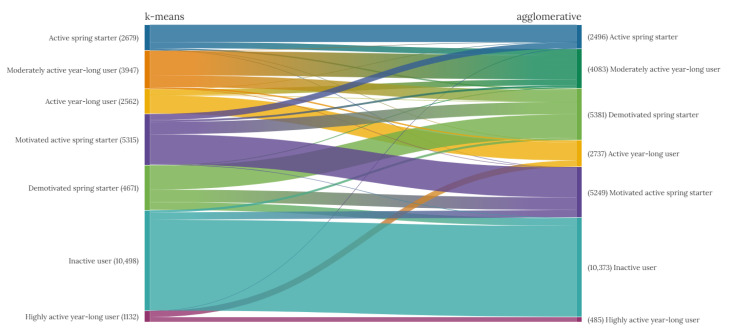
Sankey diagram of cluster agreement between k-means and agglomerative clustering methods for yearlong physical activity behaviour. The thickness the line the indicates the number of years of behaviour assigned to both the corresponding k-means and agglomerative clusters.

**Figure A4:**
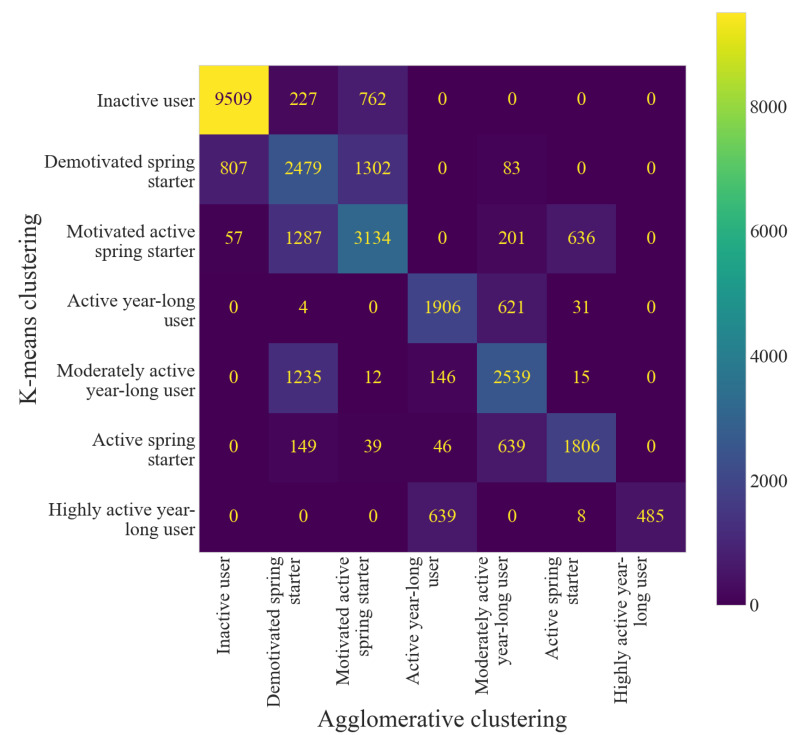
Confusion matrix outlining the agreement between the two clustering methods.

Cluster agreement and sizes, as illustrated in Figure 8, are similar between the agglomerative and k-means methods when classifying yearlong physical activity behaviour. The MRI score, reported in Table 2, indicates a high level of agreement between the cluster assignments of the agglomerative and k-means clustering analysis for yearlong activity behaviour, therefore there is a high similarity between the clustering methods. Even after adjusting for chance (AMI) and effectively controlling against increased MI due to a larger number of clusters, this remains relatively high for yearlong activity behaviour. Similarly, ARI, measuring the similarity of the yearlong cluster assignments, is also fairly high. Figure 8 demonstrates that most of the disagreement between clustering methods for yearlong activity behaviour is associated with the clusters we have labelled motivate and demotivated ‘spring starter’ user behaviours, alongside some variation in classification based on where activity intensity is ‘cut’. The full confusion matrix of the association between the clustering methods is available in Figure A4.

In terms of weekly physical activity behaviour, the MI and AMI are still fairly high indicating a good level of agreement between the k-means approach and the agglomerative clustering on the samples of weekly activity behaviour (Table 2). ARI however is no very high suggesting similarity of assignments between the two methods is not as good as for yearlong behaviour. However, some difference is expected due to applying agglomerative clustering to only a sample versus the k-means clustering being applied to the full dataset.

## 4. Cluster Characterisation

There was no significant difference in demographic distribution in the yearlong or weekly clusters as defined by the two clustering methods, hence only the cluster characterisation for the k-means clusters are presented.

### 4.1. Cluster Demographics

#### 4.1.1. Yearlong Physical Activity Behaviour Cluster Demographics

Every cluster of yearlong activity behaviour contains behaviours recorded by both male and female users, as shown in Table 3. However, male users are more likely to be clustered in the *inactive* and *highly active yearlong clusters*, whilst female users were more likely to be *moderately active* through the year or *spring starters* who either increased activity throughout the year or became demotivated. Gender is not significantly associated with belonging to the *active spring starters* or *active yearlong* clusters.

**Figure 9:**
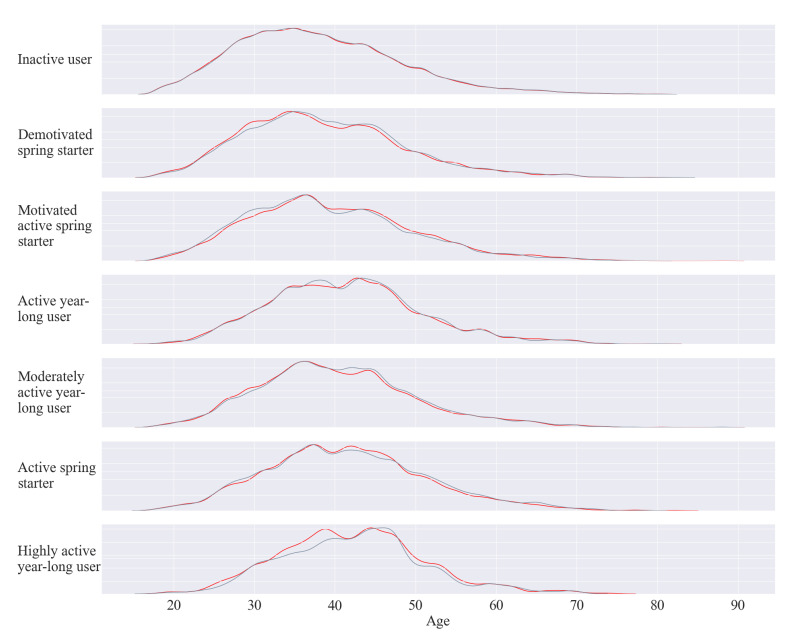
Age distribution of users in yearlong physical activity behaviour clusters (grey: k-means, red: agglomerative clustering).

In Table 3, we can see the average age of the clusters varies slightly. However, when we visualise the age distributions in the kernel density estimate plot in Figure 9 we can see different activity behaviours are associated with different age profiles. The *inactive* cluster seems to be indeterminate of age, with the age distribution similar to that of the age distribution of all users. Notably, the *highly active* cluster has the oldest average age and age distribution profile, suggesting the app may appeal to active older users, reflected in the *active yearlong* cluster. Younger age distributions are associated with the three *spring starter* clusters, with the youngest age distribution of these three clusters seemingly associated with motivated behaviour.

#### 4.1.2. Weekly Cluster Demographics

In Table 4, the proportion of weeks of activity recorded by men classified as *consistent low activity* and *consistently highly active* was significantly higher than the proportion of weeks recorded by women in these cluster. Conversely, there was a significantly higher proportion of weeks of activity recorded by women than men in the *consistently somewhat active* and *weekday active* clusters.

### 4.2. Further Physical Activity Behaviours of the Clusters

#### 4.2.1. Physical Activity Behaviour of Yearlong Clusters

**Figure 10:**
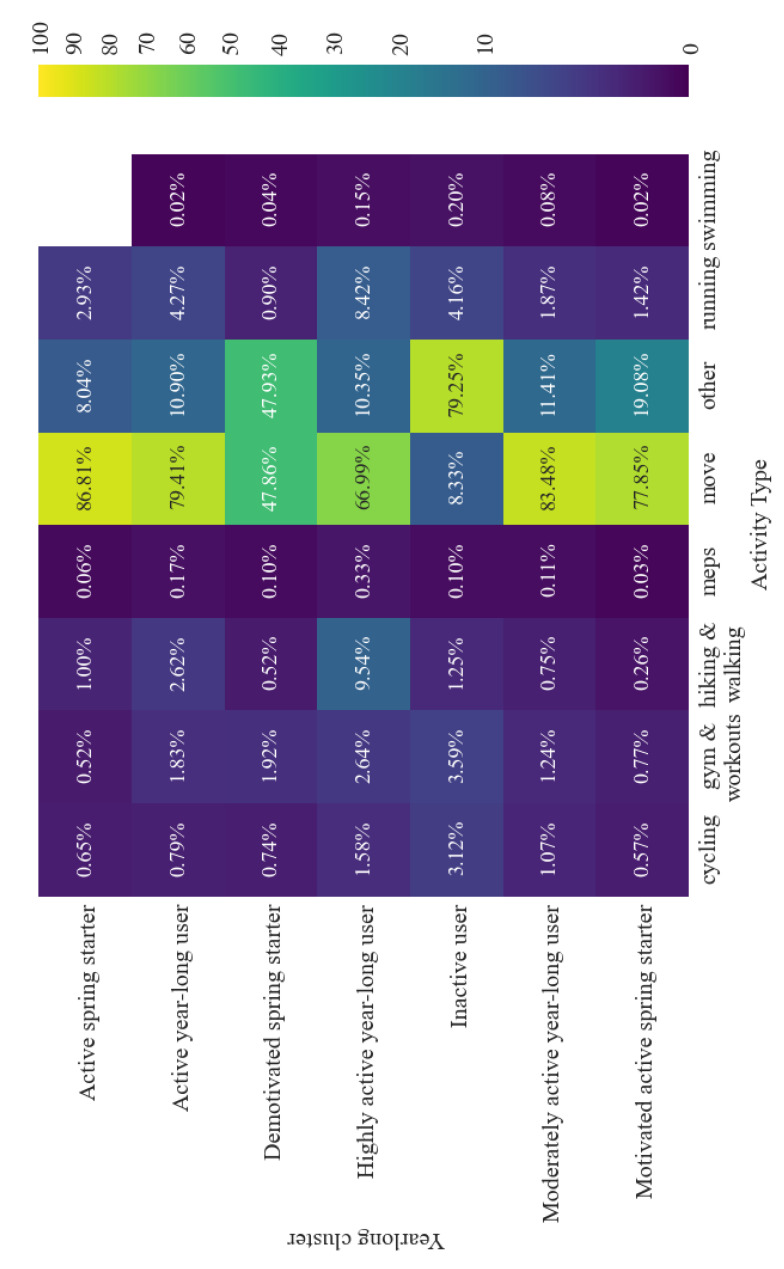
Proportion of activities recorded by each cluster by activity type.

Preference for different types of physical activity varied by cluster (Figure 10). As previously identified, ‘move’ activities are the most commonly recorded [29]. The inactive cluster, as defined by step count, subsequently has a higher percentage of cycling and gym activities, typically non-step related activities. Equally, the proportion of activities associated with a higher step count, such as running and walking activities, is highest in the highly active yearlong group. The moderately active, active, and highly active yearlong behaviour clusters show a trend of increased activity type diversity the more active the cluster.

**Figure A5:**
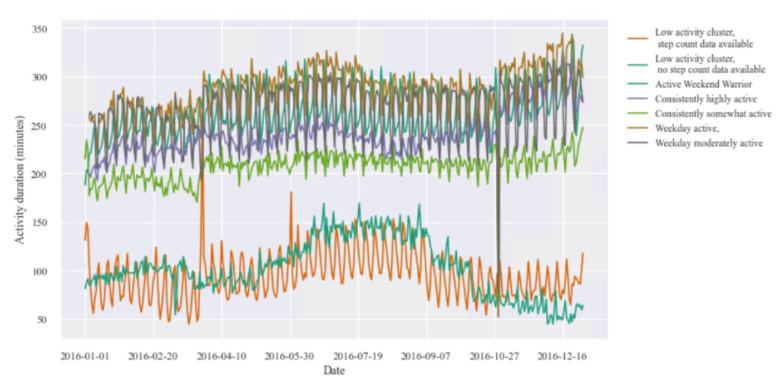
Activity duration by yearlong step count cluster, Inactive cluster disaggregated by step count data availability.

As previously identified, not all activities have an associated step-count, dependent on the app used to record the activity and activity type. Overall, 49.7% of activities recorded by the *inactive cluster* had no step count, compared to just 14.4% of activities recorded by users in other clusters. Therefore, we also looked at activity duration as a metric for activity volume by comparing activity duration between step count defined yearlong clusters and disaggregating the *inactive cluster* based on availability of step count data (Figure A5). All clusters showed a similar seasonal pattern in activity duration as seen in the step count. The *inactive cluster* with step count data showed a similar pattern in activity duration to the other clusters, but activity duration was still diminished. The *inactive cluster* members with no step-count data showed a similar activity duration behaviour to the *inactive cluster* members with a step count. However, there was more variation in mean activity duration across the cycles suggesting intermittent activity recording, e.g., recording a cycle ride a couple of times a week.

#### 4.2.2. Physical Activity Behaviour of Weekly Clusters

Table 5 depicts the number of activities and activity diversity of the clustered weekly step behaviour of the Bounts app users. Generally, increased activity diversity and number of activities a week is associated with a higher number of active minutes on average and, therefore, a greater likelihood of meeting physical activity guidelines. The *consistently highly active* week cluster has the highest average number of weekly activities and diversity in activity (average number of different activity types). This corresponds to this cluster also recording on average the most active minutes a week and therefore being more likely to meet the 150-min physical activity guideline. Conversely, the *consistently low active* cluster has the lowest activity diversity and number of activities undertaken a week. However, the *consistently low activity* weekly behaviour cluster record on average a higher number of active minutes than the somewhat active and weekday moderately active clusters, corresponding to a higher proportion of weeks recorded by the *consistently low activity* cluster meeting the WHO physical activity guideline. As previously mentioned, this is probably associated with a sub-group of the low active cluster recording activities without an associated step count. Differences between the *active weekend warrior* and *weekday active* and *moderately active clusters* in meeting physical activity guidelines seems to be based on overall difference in average step count and not on the number of activities recorded or diversity of activities undertaken.

**Figure 11:**
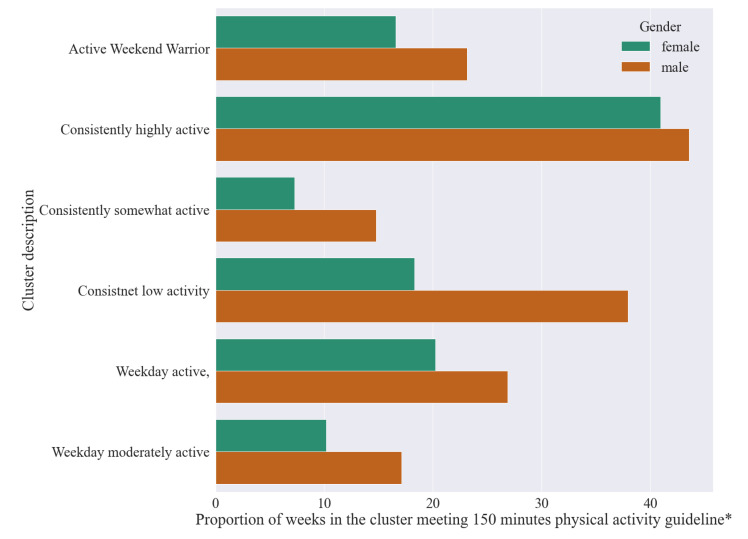
Proportion of weeks in the weekly step behaviour clusters meeting the WHO 150 min(*) of MVPA guideline, by gender.

Figure 11 depicts the proportion of weeks each cluster meets the physical activity guideline, disaggregated by gender. Male users across all clusters are more likely to meet the physical activity guidelines, with gender often playing a larger role than cluster membership in determining meeting of the physical activity guidelines. For example, *consistently somewhat* active male cluster members are more likely to meet physical activity guidelines than women in all clusters bar the consistently *highly active* cluster.

### 4.3. Cluster Interactions

**Figure 12:**
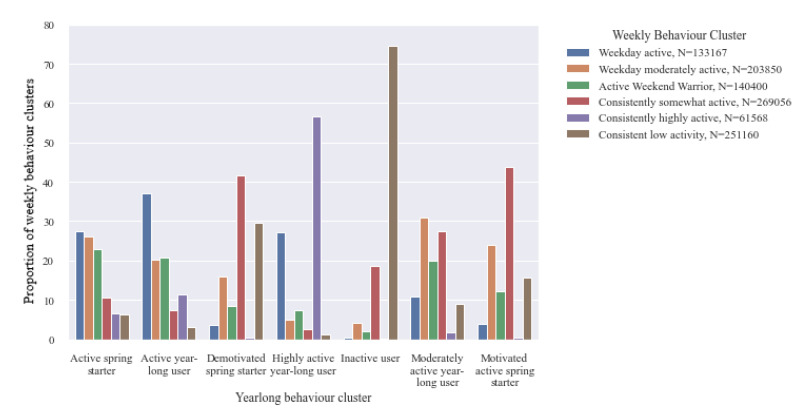
Proportion of user’s weekly physical activitybehaviour clusters contributing to their annual physical activity behaviour classification.

Figure 12 illustrates how the individual weekly behaviours recorded by users cumulatively contribute to the annual physical activity behaviour classification. For instance, *yearlong inactive* users have a high proportion of *consistent low activity* weeks. The increasingly *motivated spring starters* and *demotivated spring starters* both have a proportion of *somewhat active weeks* compared to those classified as *active spring starters*, where being *active* or *moderately active* during the week is the most common weekly physical activity behaviour.

## 5. Discussion

Through the use of secondary smartphone app data we have been able to identify seasonal and weekly clusters of daily activity behaviour in a long temporal dataset. Using step-counts as a proxy for physical activity it has been possible to detect seven unique yearlong behaviours and six distinct weekly patterns of physical activity behaviour in a group of over 30,000 smartphone users spread across the UK.

Differences between yearlong physical activity behaviour clusters can be attributed to differences in step count intensity (the moderately active, active and highlight active clusters), as well as a subset of users (32.4%) showing either motivated or demotivated activity behaviours. Female users are more likely to be in these motivated or demotivated clusters than their male counterparts, and the age distribution of these clusters tends towards younger users, whilst male and old users are more likely to be in the highly active yearlong clusters. These findings are in line with Guertler et al. who report reduced non-usage attrition risk in male and older users of a smartphone app to encourage fitness [41]. Additionally, we can link these gendered and age differences to differences in activity type choice between clustered users. Generally, wider variety and splitting of activity choice between different types is in line with the more active cluster membership, mirroring the likelihood of male users to undertake a wide variety of activities, as previously discussed by Pontin et al. [29]. Future sociodemographic stratification of yearlong activity behaviours is warranted to further understand the role seasonality plays in observed physical activity behaviour.

Within all the yearlong activity clusters, bar the demotivated cluster, we see an increase in physical activity with increasing daylight over the summer months, in line with previous findings regarding daylight saving by Pontin et al. [29]. However, there is a slight dip in activity in July and August. A July dip within overall higher levels of physical activity in the summer months has previously been identified in studies using pedometer [19], accelerometer [42], and smartphone app data analysis [22] to capture physical activity behaviour. All these studies also have the advantage of continual or high frequency repeat physical activity monitoring, allowing this behaviour to be identified. An explanation for this July dip has been attributed to a potential increase in days off from work during the July months or warmer temperatures in the Northern hemisphere discouraging individuals from being physically active [19]. Childcare and sport facility availability may also play a role with this time period corresponding to school holidays in the UK [43]. Identification of seasonal behaviour in physical activity in children has driven policy to increase activity over the summer holidays [44,45], however further investigation is required to identify what is driving these behaviours in adults to address this seasonal reduction in activity behaviour.

Despite evidenced influence of time of year and daylight on physical activity [46,47], when investigating activity behaviours seasonality is often controlled for. Limited availability of habitual data due to short data collection periods is often the driver for needing to control for seasonality. However, secondary smartphone app data present a cost-effective method to passively collect long-term physical activity data with minimal participant burden [48]. Allowing further exploration of these seasonal patterns to provide useful insight into more complex drivers of activity behaviour. Paired with detailed spatial data they also provide the opportunity to guide targeted local policy intervention to encourage physical activity.

Previous analysis of weekly behaviour in the Bounts dataset has only looked at total number of activities recorded by all users across the year [29]. By controlling for week-to-week variation and calculating the absolute deviation in the number of daily activities recorded from the weekly average across all users, weekends were lower in the number of activities recorded and had lower activity counts compared to weekdays when activity levels peaked on a Tuesday [29]. Though this is a commonly applied method, through the step-count clustering analysis we illustrate this is not a universal behaviour. Whilst some users do follow a mid-week peak step count and weekend low steps count series of behaviour (weekday active and moderately active clusters) a substantial proportion (54.9%) of user recorded weeks follow a consistent different pattern of behaviour across the week at different activity intensities and 12.8% are weekend warriors with higher activity on the weekends.

A low proportion of weekend warriors is in line with other studies, which classed between 1% to 7% of individuals as weekend warriors [13,15,46,48]. Previously we identified that app users were more likely to be from areas with a lower socioeconomic status [29], therefore this low proportion of weekend warrior users might be attributed to the socio-demographic makeup of the users. Shuval et al. identify higher-income individuals were more likely to be weekend warrior and meet the physical activity guidelines over a shorter time period [16]. Though we do not see a significant difference in the percentage of weeks classified as ‘weekend warriors’ by gender the evidence around the role of gender in weekend warrior behaviours remains mixed. Several studies have identified men were more likely to be classified as weekend warriors than women [15,49], however many studies do not explore the role of gender at all [50,51].

Pontin et al. previously have discussed the potential reasons for a low proportion of Bounts users meeting of the MVPA guidance, including the high proportion of female users who despite moving throughout the day often record activity that does not meet the threshold of MVPA [29]. By exploring the profiles of weekly behaviour we can see that weekly behaviour patterns play a prominent role in meeting MVPA guidance. In line with other research we see that ’weekend warriors’ engage in around half the amount of MVPA than consistently highly active individuals [3]. Nonetheless, gender is seemingly a stronger indication of meeting physical activity guidance than cluster membership, with male users consistently more likely to meet the guidance than female users across all clusters. Male users are also more likely to be in the more active weekly clusters in the first place. As previously reported, male users record both a higher average number of activities a day and a higher average number of different activities whilst using the app [29]. Across the week increased activity diversity and activity frequency is associated with the more active clusters and an increased number of active minutes. Increased activity variety has previously been associated with increased likelihood of meeting the WHO physical activity guidelines and increased energy expenditure in intervention studies [52,53]. However, the main body of evidence around activity variety and physical activity is in childhood not adult populations, reflected in the UK Chief Medical Officers’ Physical Activity Guidelines which recommend variety of activity for 1–18 year olds but not in adult or older adult populations [54]. Future research outside of an intervention setting needs to be completed into the role of activity type variety in meeting activity guidance.

By using a longer time period than traditional study designs we show that users do not consistently follow the same weekly behaviour patterns, recording weeks of activity that fall into several different classifications. This intra-individual variation suggests the typical seven-day data collection period will not capture the full variety of an individual’s activity behaviour [5]. Therefore, a longer time period of physical activity monitoring is needed to capture habitual behaviour. Determining when and why these changes in weekly behaviour occur warrants further investigation and could drive both development of just-in-time intra-individual interventions to increase physical activity and wider inter-individual driven policy interventions. Moreover, linking weekly behavioural pattern combinations to overall annual activity patterns helps to explain these further. For instance, being a weekend warrior seemingly does not contribute to longer term patterns of higher levels of activity. Whereas those who are active mid-week seemingly have a more consistent activity behaviour across the year. To our knowledge this is the first study to relate weekly physical activity behaviours to annual physical activity behaviour.

Using unsupervised clustering methods to identify patterns in step count we have not relied on prior assumptions to classify these behaviours [9]. Meyer et al. have previously identified patterns of use of activity trackers [55]. However, they relied upon a qualitative approach to detect patterns in the data [55]. Though the use of expertise is useful in defining patterns, this is only feasible in a smaller sample size and an inability to scale this analysis to a larger sample size limits wider generalisability to the general population [55]. Furthermore, clustering analysis is scalable and specific to the populations of interest.

Compared to previous clustering and behavioural pattern identification approaches our study is advantageous in comparing day-to-day variation in activity, using daily step counts rather than aggregate measures. Despite detailed individual level data obtained from smartphones it is commonplace that activity behaviours are aggregated and then averaged out, losing the detail or variety in observed behaviours. For instance, Althoff et al. aggregate behaviour to city level and then take the average step count at 30 min intervals [20]. Whilst these aggregate approaches are useful for population comparisons, they may well mask behaviours of population subsets which need to be understood to target policy to increase physical activity. Similarly, in the aforementioned study by McConnell et al. where they identified ten physical activity behaviours from smartphone app data, they use aggregate measures, such as percentage of time spent active to define the clusters of behaviour [9]. The temporal richness of these data is a particular advantage of using smartphone app data. Appropriate method choices need to be made to best make use of this detail physical activity behaviour information. A limitation of using step counts, despite being the most common metric, to inform the cluster names is that some activities do not have an associated step-count. Therefore, individuals that cycle or swim, activities that are more popular in male users [29], may be classed as *inactive* despite being more likely to meet physical activity guidelines. Future studies would benefit from deriving a composite measure of activity, such as METS, to better incorporate different activity types into the clustering.

The similarity between the partitioning and hierarchical clustering methods, as well as their identification of patterns identified in other settings, indicates they are suitable methods for classifying physical activity behaviours in similar datasets in the future. Nevertheless, these methods are not without their limitations. K-means clustering tends to produce uniform cluster sizes [56]. As we can see in the sizes of both the weekly and yearly clusters, unsurprisingly behaviours are not equally distributed throughout the population. Possibly explaining the disagreement in clustering method assignment to the motivated and demotivate spring starter clusters. Therefore, using k-means may mask smaller clusters of behaviour. In the future, studies could use different cluster initiation techniques to better detect the smaller cluster centroids [57]. Data volume limited the ability to apply agglomerative clustering to the weekly physical activity behaviour data as the analysis was costly in terms of computational memory and time. Though we addressed this via repeat agglomerative clustering on subset data and subsequent identification of patterns in overall dataset. This issue can also be addressed by use of high-performance computing services or cloud computing [26], however these have associated monetary costs and skill requirements. Moreover, data security of any computing service used must be considered. Thus, these clustering method limitations are important with respect to method selection.

We only included investigation of hierarchical and partitioning methods as density methods, for instance density-based spatial clustering of applications with noise (DBSCAN), were expected to perform badly due to the difference in densities of the observed temporal patterns and closeness of the potential clusters [30]. This was confirmed in preliminary exploratory analysis. Future investigation could expand the clustering methodologies employed. For instance, the issue of differentiating density in DBSCAN clustering could be addressed using hierarchical DBSCAN. Dynamic time warped (DTW) k-means was also considered, using optimal alignment instead of Euclidean distance between the sequences of behaviour [58]. However, DTW k-means unifies the same pattern with time shifts in the data. For instance, an increase in magnitude of behaviour across two days of the week would be treated the same by the DTW algorithm if the same level of magnitude increase occurred mid-week or on the weekend. As we were interested in the time and magnitude of activity behaviours DTW was not deemed a suitable clustering method. Future studies looking to identify general activity patterns of behaviour could however benefit from application of DTW k-means. Fuzzy C-Means is another possible future method to investigate, similar to k-means clustering but behaviours do not have to belong to a single cluster. This would help detect the borderline behaviour for instance when we saw disagreement over k-means and agglomerative clustering due to different apparent cut-off points in step count intensity. However, the interpretability of these fuzzy clusters may limit wider applicability of identified patterns.

Though there are many benefits to using secondary smartphone app data, the nature of using app data designed originally to encourage physical activity has some limitations, in particular the potential of app premise to influence recorded activity behaviour [28]. Evidence regarding the influence of apps on long-term activity behaviour however remains mixed. In their meta-analysis Romeo et al. found that smartphone apps produced a non-significant increase in participant’s daily average step count compared to controlled conditions [59]. Additionally, they report that programs longer than 3 months, similar to Bounts app usage, were less effective in increasing physical activity than shorter programs [59].

Previous investigations have shown that Bounts app attracts a much higher proportion of female users [29]. This is contrary to previous studies using physical activity smartphone app data which have been found to over represent young male users [9,60,61]. Thus, any researchers looking to replicate this analysis in a different smartphone app data source would need to also consider the socioeconomic and demographic profile of the app users. Additionally, we use a single measure to determine whether WHO physical activity guidance was met which might explain the overall low proportion of users classed as sufficiently active, future research would benefit from multiple activity specific measures to quantify physical activity [62]. Future work would also benefit from multi-year investigation. This would allow confirmation of the seasonal observed patterns and allow identification of any app-based influences on activity behaviour. For instance, we see a larger number of sign-ups around the beginning of British summertime and have shown that an increase in activity occurs independent of sign-up date. Repeat year investigation would allow us to identify whether this sign-up phenomenon occurs annually or is a one off linked to app promotional activity. Additional demographic detail, such as occupation, ethnicity, health status and family composition, would also aid in disentangling these behavioural patterns further. However demographic detail must be weighed against maintaining individual anonymity in secondary smartphone app data sources [28].

## 6. Conclusions

Secondary smartphone app data proves to be a useful tool to capture temporal patterns in physical activity behaviour. The size and richness of these app data lend themselves to detailed pattern analysis. Exceeding the typical seven-day study period we identify that individuals can belong to several groups of weekly behaviour, suggesting that this shorter time period is not suitable to capture habitual activity behaviours. Through the application of machine learning methods, we can move away from aggregate measures of physical activity to identify and better understand these temporal variations in physical activity behaviour.

## Figures and Tables

**Table 1:** Clustering method description.

Clustering Algorithm	Class of Algorithm	Example of Application to Seasonal Physical Activity Data
k-means	Partitioning	Yearly activity behaviours are partitioned into a predefined number of clusters, minimising within cluster difference (within cluster sum of squares) and maximising between cluster difference. Initially randomly positioned centroids are used and each year of activity behaviour assigned to its nearest centroid, the mean of the centroids is calculated and the process is iteratively repeated until the centroids do not change [30]. These are then the defined clusters.
Agglomerative	Hierarchical/ linkage	Initially each unique year of activity behaviour is treated as its own cluster, the most similar clusters are then grouped together to form larger clusters. The point at which we chose to stop cluster merging and examine the remaining cluster is determined using a dendrogram.

**Table 2:** Model evaluation metrics for yearlong physical activity step behaviours.

Validation Metric	Seasonal Physical Activity Behaviour Clusters	Weekly Physical Activity Behaviour Clusters
Mutual Information score (MI)	0.992	0.879
Adjusted mutual information score (AMI)	0.569	0.523
Adjusted Rand Index (ARI)	0.577	0.413

**Table 3:** Demographic characteristics of k-means yearlong physical activity behaviour clusters. Bold font indicates a statistically significant associating, significantly higher proportions are shaded grey.

Cluster Description	Number of Users	Proportion of		*p*	Mean Age
		Female Users	Male Users		
Active spring starter	2679	8.67	8.81	0.730	41.46
Active yearlong	2562	8.28	8.44	0.686	41.48
Demotivated spring starter	4671	15.91	12.57	**<0.05**	38.49
Highly active yearlong	1132	3.31	4.93	**<0.05**	42.21
Inactive	10,498	32.21	40.61	**<0.05**	37.81
Moderately active yearlong	3947	13.32	11.06	**<0.05**	40.22
Motivated spring starter	5315	18.31	13.58	**<0.05**	39.74

**Table 4:** Gender and activity behaviour characteristics of k-means weekly physical activity behaviour clusters. Bold font indicates a statistically significant associating, significantly higher proportions are shaded grey.

Cluster Description	Number of User Weeks	Proportion of Weeks Recorded by		*p*
		Female Users	Male Users	
Active Weekend Warriors	140,400	13.28	13.16	0.160
Consistently highly active	61,568	5.25	7.8	**<0.001**
Consistently somewhat active	269,056	26.83	20.37	**<0.001**
Consistent Low Activity	251,160	21.88	30.18	**<0.001**
Weekday active	133,167	12.47	12.93	**<0.001**
Weekday moderately active	203,850	20.29	15.56	**<0.001**

**Table 5:** Physical activity characteristics of weekly physical activity behaviour clusters.

Cluster Description	Average Number of Activities Undertaken per Week	Average Number of Different Activity Types Undertaken	Average Active Minutes per Week	Proportion of Cluster Weeks Meeting MVPA Guidelines *
Active Weekend Warriors	9.46	1.4	107.7	19.9%
Consistently highly active	17.5	1.9	303.8	42.3%
Consistently somewhat active	7.3	1.2	59.0	11.0%
Consistent Low Activity	4.2	1.2	145.6	28.1%
Weekday active	11.2	1.5	137.2	23.5%
Weekday moderately active	8.4	1.3	72.6	13.7%

* 150 min of moderate to vigorous physical activity as recommended by the WHO.

## Data Availability

The data presented in this study are available on request from the Consumer Data Research Centre (https://public.cdrc.ac.uk/dataset/active-inspiration-\T1\textendashactivity-data, accessed on 1 February 2021). Access is available via a remote service with registration and project approval requirements. The data are not publicly available due to access being restricted due to license conditions.

## Abbreviations

The following abbreviations are used in this manuscript:

WHO	World Health Organisation
MVPA	Moderate to Vigorous Physical Activity
DBSCAN	Density-Based Spatial Clustering of Applications with Noise
DTW	Dynamic Time Warped
ARI	Adjusted Rand Index
MI	Mutual Information
AMI	Adjusted Mutual Information
CDRC	Consumer Data Research Centre
